# Supplementary material for: Bioinformatic Reconstruction and Analysis of Gene Networks Related to Glucose Variability in Diabetes and Its Complications
Source: Int J Mol Sci. 2020 Nov 18;21(22):8691. doi: 10.3390/ijms21228691 (PMC7698756; doi:10.3390/ijms21228691)
Supplement: Supplementary file 1 [file ijms-21-08691-s001.zip › ijms-982039-revised-supplementary/Figure S3.pptx]

## Slide 1
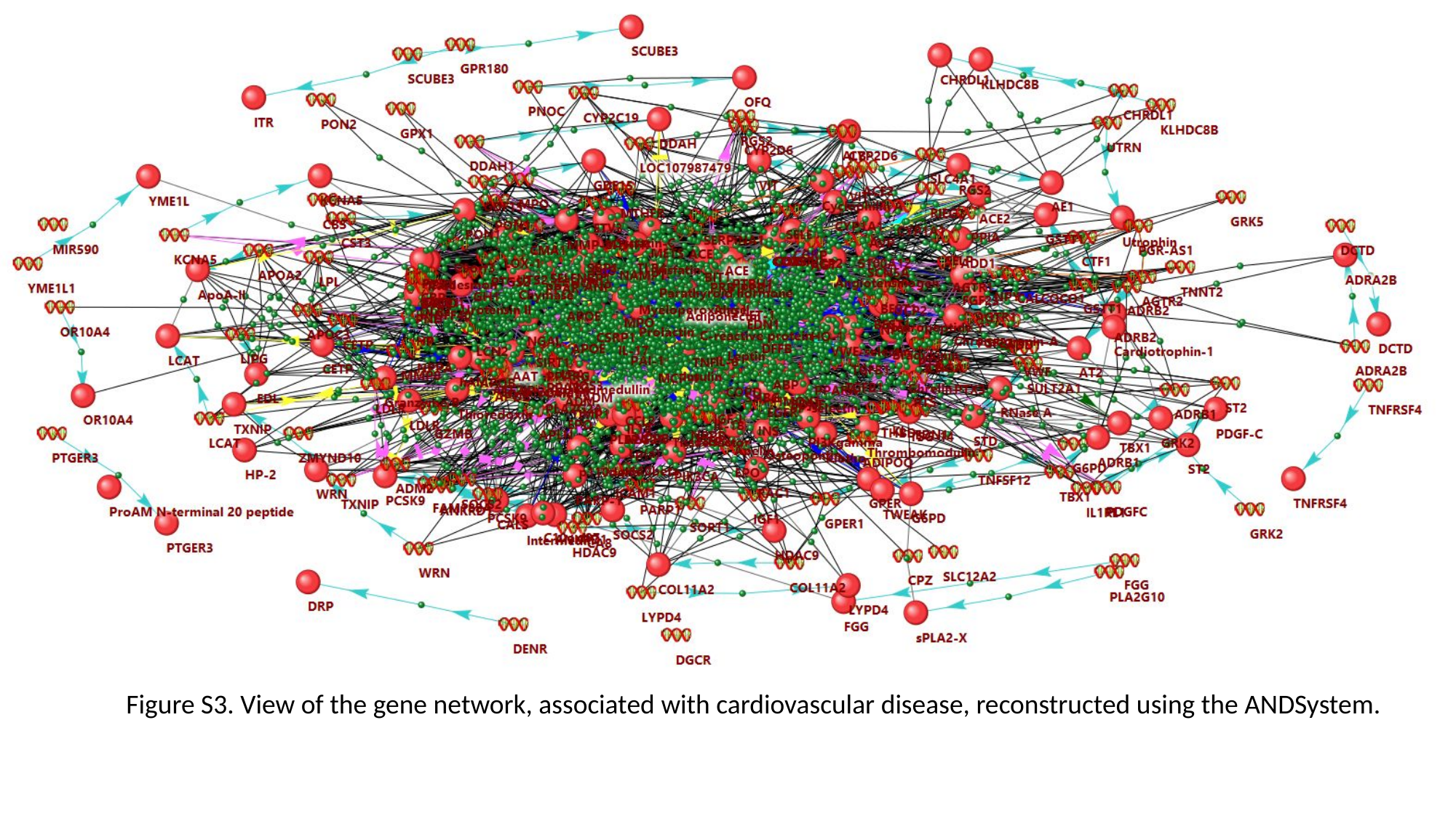

Figure S3. View of the gene network, associated with cardiovascular disease, reconstructed using the ANDSystem.
